# Effect of Noble Metal Addition on the Disorder Dynamics of Ni_3_Al by Means of Monte Carlo Simulation

**DOI:** 10.3390/ma13214832

**Published:** 2020-10-29

**Authors:** J.J. Ramos-Hernandez, C.D. Arrieta-Gonzalez, J.G. Chacon-Nava, E. Porcayo-Palafox, M. Sanchez-Carrillo, J.P. Flores-De los Rios, G.K. Pedraza-Basulto, S.E. Diaz-Mendez, J. Porcayo-Calderon

**Affiliations:** 1 Facultad de Quimica, Universidad Nacional Autonoma de Mexico, Edificio B, Cd. Universitaria, Mexico City 04510, CDMEX, Mexico; josejuanramos@gmail.com; 2 Instituto de Ciencias Físicas, Universidad Nacional Autónoma de México, Avenida Universidad s/n, Cuernavaca 62210, MOR, Mexico; 3 Tecnológico Nacional de Mexico-Instituto Tecnológico de Zacatepec, Calzada Instituto Tecnológico 27, Zacatepec 62780, MOR, Mexico; cdaglez@gmail.com; 4 Centro de Investigación en Materiales Avanzados (CIMAV), Miguel de Cervantes 120, Complejo Industrial Chihuahua, Chihuahua 31136, CHIH, Mexico; jose.chacon@cimav.edu.mx; 5 CIICAp, Universidad Autónoma del Estado de Morelos, Avenida Universidad 1001, Cuernavaca 62209, MOR, Mexico; porcayo@gmail.com; 6 Tecnológico Nacional de Mexico-Instituto Tecnológico de Chihuahua, Av. Tecnologico 2909, Chihuahua 31130, CHIH, Mexico; msanchezc@itch.edu.mx (M.S.-C.); jpfloresr@itch.edu.mx (J.P.F.-D.l.R.); 7 Facultad de Ingenieria, UNACAR, Ciudad del Carmen 24180, CAM, Mexico; gpedraza@pampano.unacar.mx (G.K.P.-B.); sdiaz@pampano.unacar.mx (S.E.D.-M.)

**Keywords:** intermetallics, order/disorder transformation, site occupancy, Monte Carlo simulation, pair correlation function, ordering energies

## Abstract

In this work, the effect of the addition of noble metals on the order–order disorder process of the L12 structure corresponding to the intermetallic Ni_3_Al is analyzed. Stoichiometric, nonstoichiometric, and quasi-binary compositions doped with noble metals such as Ag, Au, Pd, and Pt (1 at%) were analyzed. It was observed that depending on the composition, there is a modification in the activation energies calculated from the two time constants that characterize the disorder process. The statistic of atomic jumps was typified based on the configuration of the window to be crossed and, with this, it was identified that the origin of the negative activation energy of the long disorder process is due to an increase in the corresponding energy of the Al_Al-Ni_ jump through unnatural windows.

## 1. Introduction

Nickel aluminides, such as NiAl and Ni_3_Al, are potential materials for high-temperature structural applications. Its physicochemical and mechanical properties are attributed to the high level of ordering both in the short and long range of its lattice, and to the order–order and order–disorder transitions that occur in these intermetallics [1].

**Figure 1:**
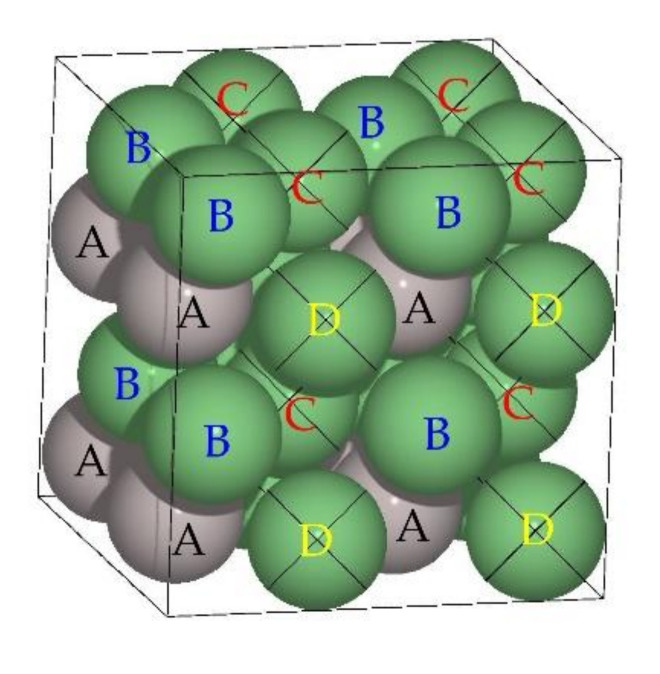
Atomic distribution in the L_12_ supercell corresponding to the Ni_3_Al, and the crossed-out atoms make up the layer formed only by Ni atoms.

The Ni_3_Al lattice structure can be visualized as four interpenetrated sub-lattices, the atomic distribution of which is such that each atom is surrounded by 12 atoms that make up the first coordination layer commonly called near neighbors (NN). The interpenetrated sub-lattices can be visualized in Figure 1, the atoms corresponding to the Al sub-lattice are identified with the letter A and those of Ni with the letters B, C and D. For Ni atoms, the first coordination layer consists of eight Ni and four Al atoms, while for Al atoms, the coordination layer consists of only Ni atoms. Another important characteristic is that they form a combination of layers with different compositions, one of 50%Ni-50%Al (more stable layer) and another of 100%Ni (Figure 1); this characteristic is fulfilled in the crystallographic planes (001), (010), and (100) [2].

As the temperature increases, changes occur in the position of the atoms in the Ni_3_Al structure, forming new configurations that are governed by the ordering kinetic rules, which generates a modification in the long- and short-range order parameters (LRO and SRO) until equilibrium is reached. The LRO parameter remains elevated until a temperature of 800 °C from which it decreases until reaching a value of zero at 1475 °C, 100 °C higher than the melting temperature, and is called the order–disorder transition temperature. Due to the high value of LRO, atomic mobility and self-diffusion are controlled by atomic migration into an almost perfect superstructure, in which the predominant mechanism consists of elemental jumps to neighboring vacancies [3] rather than a direct exchange between atoms [4].

The ordering kinetics of Ni_3_Al has been investigated by resistometry [5,6], and modeled during the last decades by means of Monte Carlo kinetic simulation [3,7,8,9,10,11,12], where it has been defined that the jump probability of an atom depends on the change in energy state between its initial and final position (determined by the interaction energies between pairs).

When alloying elements are added to the Ni_3_Al structure, the preferential site of substitution can influence its properties. The preferential site of ternary atoms is governed preferentially by the electronic structure rather than by the size factor [13], and three cases are possible [14], for example, preference for Ni sites, preference for Al sites, and/or combined preference, the latter depending on composition and/or temperature. Due to these conditions, it is possible that there are new energies of interaction between pairs and window configurations that affect the dynamics of atomic mobility and, thus, the properties of the intermetallic.

Into the Ni_3_Al compound, the Ni atoms always diffuse faster than the Al atoms, this is due to the fact that Ni atoms can transit over the Ni sub-lattice in a stable manner without changing the ordering level; however, the movement of the Al atoms implies an alteration of the ordering level because it does so through a Ni vacancy [15]. Therefore, considering that the diffusion coefficients of the Ni and Al are affected when an alloying element occupies its respective sub-lattice [16], and as the noble metals (Ag, Au, Pd, and Pt) tend to occupy the sites of Ni atoms [17], a change in the atomic dynamics can occur and the diffusion coefficients into the intermetallic can be modified.

Therefore, a Monte Carlo simulation analysis applied to the intermetallic Ni_3_Al, its quasi-binary compositions (slightly rich in Ni or Al), and its doping with noble metals (1% at Ag, Au, Pd, or Pt) can reveal the changes in its ordering kinetics. This study reports the results of the Monte Carlo simulation of the relaxation mechanism of the L_12_ superstructure disorder due to the variation in its composition and noble metal doping.

## 2. Methodology 

### 2.1. Monte Carlo Simulation Algorithm

In this study, based on the Glauber–Metropoli algorithms, a Monte Carlo (MC) simulation algorithm was coded, tested, and compared with previous results for the Ni_3_Al superstructure. The simulations were run in a canonical assembly with 65,536 (32 × 32 × 64) cell units with a total of 262,144 spaces occupied by Ni and Al atoms in a stoichiometric ratio for Ni_3_Al. The initial distribution of the atoms in the lattice corresponds to that of a perfect arrangement (assuming a temperature of 0 K). Considering that the concentration of vacancies in Ni_3_Al is low (5 orders of magnitude less than in NiAl [3,18], only one vacancy was introduced to the system by removing an atom at a random location. As the vacancy concentration in the system will remain constant, the results are representative only of the atomic migration phenomenon [19]. It is assumed that the lattice is free of defects such as dislocations and grain boundaries, and that it is rigid so that vibrations or elastic forces are not considered. Therefore, only the configuration details are taken into account by the model, where the energy states are described in terms of local occupation variables [1]. Periodic boundary conditions were imposed on the assembly.

In order to simulate the quasi-binary compositions, 10,485 atoms (1 atomic %) of the Ni or Al sublattices were replaced by atoms in excess of Ni, Al, and noble metals (as the case may be), according to the previously described canonical assembly. The distribution of excess Ni, Al, and noble metal atoms was homogeneous through the Ni or Al sublattices, in such a way that each noble metal atom was surrounded by two atoms of the same noble metal.

Each MC simulation cycle is considered as a unit of time and consists of the random selection of an atom from the first coordination layer of the vacancy (the near neighbors). The jump of the atom toward vacancy is allowed or denied by comparing a random number (*R[0,1]*) and the probability of jump (*P*). If *R* < *P*, the jump is allowed, otherwise, it is denied. These alternatives can be extended by considering the type of atom that jumps and the direction in which it jumps, the type of window it passes through, etc. However, the use of the MC simulation cycle is commonly used in many works related to the subject. Regardless of the time unit selected, the long-range sort level and the time constants that characterize it may vary. Leitner et al. [20] mention how difficult it is to correlate an MC simulation cycle with a real time interval and found that there is a linear correlation to them. Similarly, Yaldram et al. [4] mention that there is a direct relationship between real time and MC simulation cycles. The jump probability, *P*, is calculated according to the equation:

(1) $$P=\prod_{i\to j}=\frac{e^{-\frac{\Delta E}{kT}}}{1+e^{-\frac{\Delta E}{kT}}},$$

where Δ*E* = *E_j_* − *E_i_* is the configurational energy change, and *k* and *T* are the Boltzmann constant and absolute temperature, respectively. The configurational energy change is calculated with the following formula:

(2) $$E_{i}=\sum_{n,m}E_{i-m}^{\left(n\right)},$$

where *n* is the number of the coordination shell, and *E*_i-m_ is the energy corresponding to the interaction between pairs of atoms *i* and *m*. In this stage, the Hamiltonian model of Ising is introduced, in which the interaction energies between possible pairs are described by the following equations considering the first and second coordination layer [19,21]:

(3) $$E_{Ni-Ni}^{\left(n\right)}+E_{Al-Al}^{\left(n\right)}-2E_{Ni-Al}^{\left(n\right)}=W_{\left(Ni,Al\right)}^{\left(n\right)},$$

(4) $$E_{Ni-Ni}^{\left(n\right)}+E_{X-X}^{\left(n\right)}-2E_{Ni-X}^{\left(n\right)}=W_{\left(Ni,X\right)}^{\left(n\right)},$$

(5) $$E_{Al-Al}^{\left(n\right)}+E_{X-X}^{\left(n\right)}-2E_{Al-X}^{\left(n\right)}=W_{\left(Al,X\right)}^{\left(n\right)},$$

where *E* is the interaction energy between the atoms, *W* is the ordering energy, *n* is the number of the coordination layer (*n* = 1, 2), and *X* denotes the ternary alloying element (Ag, Au, Pd, and Pt). The ordering energies for the Ni-Al atoms (parameters for Equation (3)) have been established as $W_{\left(Ni-Al\right)}^{\left(1\right)}=0.035$ and $W_{\left(Ni-Al\right)}^{\left(2\right)}=-0.0165$, also considering $E_{Ni-Ni}^{\left(n\right)}=2E_{Al-Al}^{\left(n\right)}$ [8,12]. These values have been considered because, with them, the vacancy is preferably established in the Ni sub-lattice, as has been reported [10,22].

In this work, the interaction energies between the noble metals, $E_{X-X´}^{\left(n\right)}$, were established as 1/6 of the vacancy formation energy for each of the noble metals in their pure state [22,23,24]. The interaction energy between Ni and a noble metal, $E_{Ni-X´}^{\left(n\right)}$, was obtained from the calculation presented by Ruban and Skriver [13], and because the interaction between Al and a noble metal, $E_{Al-X´}^{\left(n\right)}$, cannot be established in a simple way, it was considered for all cases $E_{Al-X}^{\left(n\right)}=E_{Ni-Al}^{\left(n\right)}$, which will allow for direct evaluation of the effect of the interaction between Ni-noble metal. As there is only one vacancy in the assembly, the model does not consider the interaction between them.

### 2.2. Monitored Parameters

The simulations were carried out for each composition-configuration at the temperatures of 900, 1050, 1200, 1400, and 1650 K. Each simulation consisted of 50 × 106 cycles, and at every 100,000 cycles, the ordering parameters and jump statistics were recorded.

#### 2.2.1. Long-Range and Short-Range Order Parameters

The Bragg–Williams long-range order parameter (LRO) as a function of time characterizes the formation of Ni antisites and is determined by means of the following equation:

(6) $$\eta (t)=1-\frac{N_{Ni}^{\left(Al\right)}}{0.75\times N^{\left(Al\right)}},$$

where *N*^(Al)^ is the number of spaces in the Al sub-lattice and $N_{Ni}^{\left(Al\right)}$ is the number of Ni antisites (Ni atoms in a space of the Al sub-lattice).

The short-range ordering parameter is defined as the average number of Al antisites found in the first coordination layer of an Ni antisite [6,25]. In this work, the antisite pair correlation parameter (APC) was used according to the following equations [10]:

(7) $$APC_{Al-Ni}=\frac{N_{nn}Al^{\left(Ni\right)}}{N_{Ni}^{\left(Al\right)}},$$

where *N_nn_Al*^(Ni)^ represents the number of Al antisites found in the first coordination layer of each Ni antisite, and *N_N_i^(Al)^* is the total number of Ni antisites.

In order to characterize the affinity that Ni and Al antisites have toward noble metals, it is necessary to define a parameter similar to *APC*. This was done considering the ordering that exists in the first coordination layer of the noble metal atoms when they occupy the Ni and Al positions, as expressed by the following equations:

(8) $$APC_{Ni-X}=\frac{N_{nn}Ni^{\left(Al\right)}}{NX^{\left(Ni\right)}},$$

(9) $$APC_{Al-X}=\frac{N_{nn}Al^{\left(Ni\right)}}{NX^{\left(Al\right)}},$$

(10) $$APC_{X-X}=\frac{N_{nn}X^{\left(Ni,Al\right)}}{NX^{\left(Ni,Al\right)}},$$

where *APC_Ni-X_* is the affinity of Ni antisites to noble metal (X) atoms located at Ni sites, *APC_Al-X_* is the affinity of Al antisites to noble metal (X) atoms located at Al sites, and *APC_X-X_* is the affinity of the noble metals for each other.

#### 2.2.2. Jump Windows

In an L_12_ binary structure, an atom can move through the Ni and Al sublattice by jumping to a vacancy in three possible directions, Ni → Ni, Ni → Al, and Al → Ni. Each jump is always carried out through a window of four atoms, which can have seven different configurations [20]; however, when a third element is considered, the number of possible configurations increases to 27.

**Figure 2:**
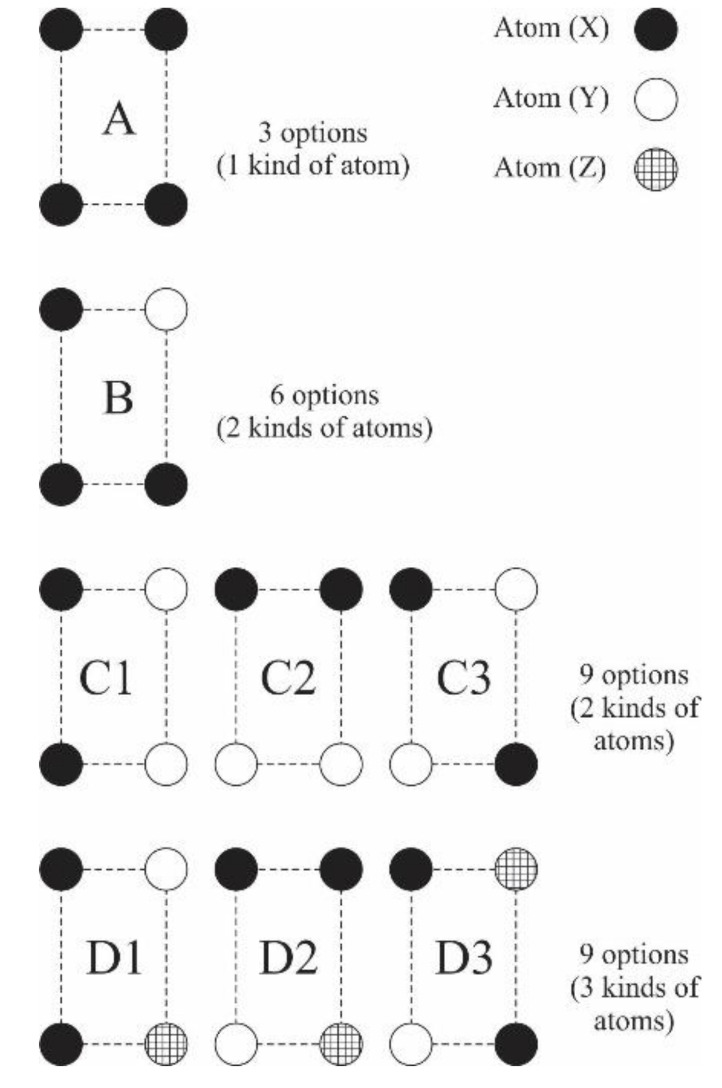
Geometrically independent window configurations, including configurations for cases C and D.

Figure 2 shows all the possible window configurations, classifying them according to the amount and type of atoms that constitute them as A, B, C, and D, also considering a subclassification with numbers from 1 to 3 for configurations C and D with the purpose of differentiating them geometrically. Configuration A contains atoms of a single element, configurations B and C contain atoms of two elements, and configuration D contains atoms of three different elements. Configurations C and D each have three sub-configurations because there are different positions of atoms in the arrangement that are geometrically independent.

In order to systematically identify a particular window configuration, the following nomenclature will be used: Letter and number of classification (and sub-classification as the case may be), followed by a list between parentheses and separated by commas of the elements contained in the window, sorting them from highest to lowest with respect to the number of times it appears in the window, or if two elements appear the same number of times, it is sorted in alphabetical order. For example, the standard windows in a fully ordered canonical array would be A(Ni) and C1(Al, Ni). The window configuration with three types of elements would be D1(Al, Ni, Pt), which is a similar configuration to C1(Al, Ni) where one Pt atom has replaced one of Ni.

#### 2.2.3. Jump Statistics

In each simulation cycle, the type of atom, the window configuration, the direction, and if the jump was allowed or denied were recorded.

## 3. Results and Discussion

In all simulations, the number of Ni and Al antisites formed or removed are the same regardless of the initial presence of antisites as in the case of nonstoichiometric compositions. This is an indication that the formation of both types of antisites is consecutive, which has been mentioned in other investigations [10].

### 3.1. Evolution of the LRO Parameter

**Figure 3:**
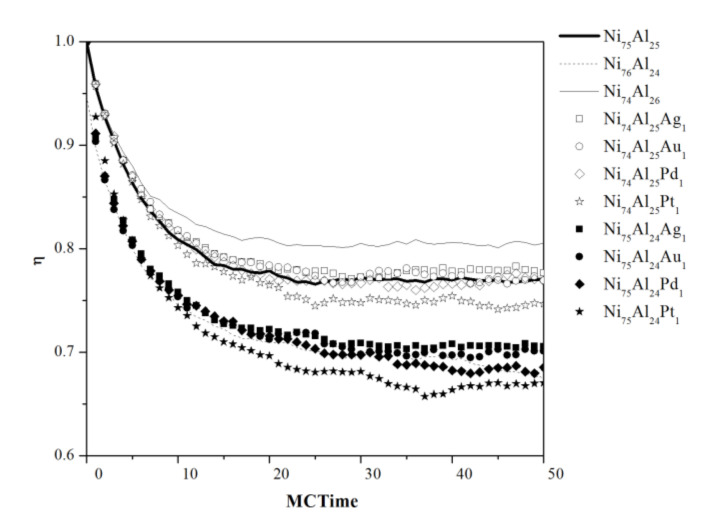
Disorder isotherms simulated at 1650 K.

Figure 3 shows the relaxation isotherms at 1650 K as a function of the simulation cycles for all the compositions studied, which is representative of the general behavior at all temperatures. It can be observed that there are significant differences between the ordering level of the nonstoichiometric formulations with respect to the stoichiometric formulation, where for the Ni_74_Al_26_, it is higher, and for the Ni_76_Al_24_, it is lower; this behavior has been mentioned in previous research by other authors [26]. The lower ordering level of the sample with formula Ni_76_Al_24_ is due to stoichiometric restrictions, that is, the Ni atoms must necessarily occupy sites corresponding to Al, which implies a lower ordering level (less than 1) at the beginning of the simulations. In compositions containing noble metals with the Ni_74_Al_25_X_1_ formula, it is similar to that of the stoichiometric composition (Ni_75_Al_25_), while the compositions with the Ni_75_Al_24_X_1_ formula have a level of ordering similar to that of the Ni_76_Al_24_ composition. It should be noted that the composition containing Pt shows a slightly lower ranking level, compared to the other compositions containing noble metals. This behavior prevails in all the temperatures studied.

**Figure 4:**
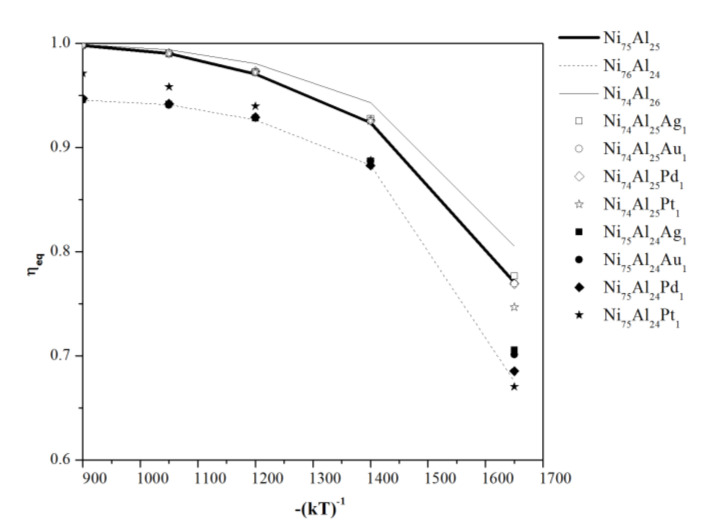
Long-range parameter at equilibrium (η) as a function of temperature for stoichiometric Ni_3_Al, Ni-rich compositions, Al-rich compositions, and noble metal-doped compositions.

Figure 4 shows the ordering level at equilibrium, (*η_Eq_*), for all compositions and temperatures analyzed. In general, it is observed that there is no significant variation between compositions (Ni_75_Al_25_-Ni_74_Al_25_X_1_) and (Ni_76_Al_24_-Ni_75_Al_24_X_1_); however, at 1650 K, the Ni_75_Al_24_X_1_ composition doped with Ag, Au, and Pd show a slightly higher ordering level with respect to that of Ni_76_Al_24_. In particular, at temperatures lower than 1400 K, the Ni_75_Al_24_-Pt_1_ sample shows a higher ordering level with respect to that of Ni_76_Al_24_; and the Ni_74_Al_25_Pt_1_ composition shows a lower equilibrium ordering level with respect to the stoichiometric Ni_75_Al_25_ composition.

**Figure 5:**
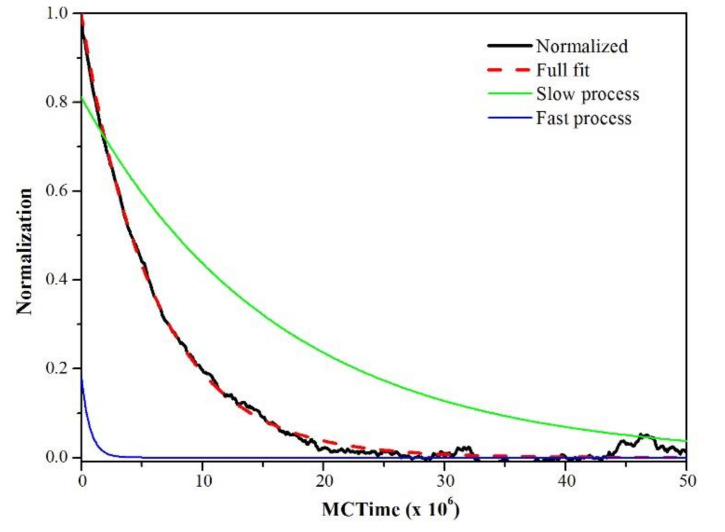
Normalization of the LRO parameter to 1200 K and fit with Equation (11). The terms that contain the time constants for the slow and fast processes are presented (right side of Equation (11)).

The disorder mechanism consists of two parallel processes with different time constants [27,28]; a fast process (short period of time) and a slow process (long period of time). These processes can be evaluated by means of the following equation:

(11) $$\frac{\eta (t)-\eta_{Eq}}{\eta (0)-\eta_{Eq}}\approx e^{\left(-t/\tau_{s}\right)}+(1-C)\times e^{\left(-t/\tau_{l}\right)},$$

where *η_Eq_* is the order parameter at equilibrium, *C* is a contribution factor (*C*[0,1]), and τ*_s_* and τ*_l_* are the relaxation times of the fast and slow processes, respectively, and must meet the condition τ*_l_* > τ*_s_*. There is a solution set (*η_Eq_*, C, τ*_l_*, τ*_s_*) that fits Equation (11) with the experimental data, which was determined by the method of least squares. As an example, Figure 5 shows the normalization of the ordering level of the sample Ni_3_Al at 1200K based on equation (11), where the contribution of the fast and slow processes can be visualized, in addition, the precision adjustment. Some authors have reported that the activation energy of the fast process does not have an adequate correlation with temperature; this has been attributed to the lack of accuracy in its determination [4]. There are various behaviors between the values of τ*_s_* and τ*_l_* [28].

**Figure 6:**
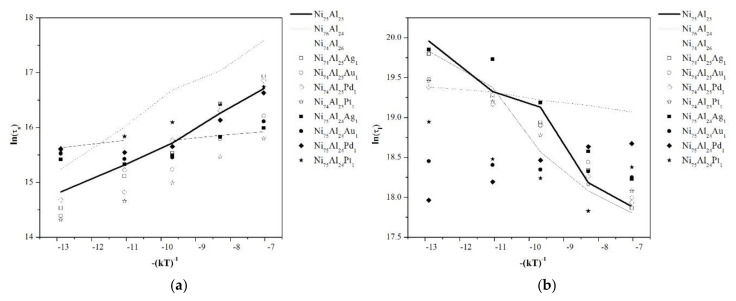
Arrhenius graphs that characterize the processes of disorder: (**a**) Fast process (τ_s_), (**b**) slow process (τ_l_).

There are several ways to present the Arrhenius equation [29], which can generate controversy in the interpretation of activation energy values [30]. Figure 6a shows the Arrhenius graph for the fast disorder process, where it is observed that the time constant (ln(τ_s_)) of each composition differs from each other by the ordinate to the origin of the lines. Compositions Ni_75_Al_25_ and Ni_76_Al_24_ have activation energies of 0.3233 and 0.3955 eV, respectively, while composition Ni_74_Al_26_ has a value of 0.0469 eV, the lowest value of all cases. The activation energies of the samples with composition Ni_74_Al_25_X_1_ are 0.4195, 0.3166, 0.3734, and 0.2582 eV for Ag, Au, Pd, and Pt, respectively. Compositions Ni_75_Al_24_X_1_ doped with Ag, Au, and Pd present an activation energy similar to that of the Ni_74_Al_26_ sample between 900 and 1200 K, and at higher temperatures, the activation energies are 0.1719, 0.1796, and 0.2767 respectively. Composition Ni_74_Al_25_X_1_ doped with Pt showed an activation energy of 0.2258 eV.

Figure 6b shows the Arrhenius plot for the slow disorder process. It is observed that compositions Ni_75_Al_25_, Ni_74_Al_26_, all those of form Ni_74_Al_25_X_1_, and those doped with Ag and Pt of the Ni_74_Al_25_Pt_1_ form have a negative activation energy between −0.2989 and −0.3691 eV, and their time constants (τ_l_) are similar. However, the Ni_74_Al_25_Pt_1_ sample presents a lower time constant, in addition to a change in activation energy from 1400 K to a value of 0.4383 eV. Compositions Ni_76_Al_24_ and Ni_74_Al_25_XAu_1_ have an activation energy of −0.0545 and −0.0333 eV, respectively, and their time constants (τ_l_) differ by almost an order of magnitude. Composition Ni_74_Al_25_Pd_1_ has a positive activation energy of 0.1293 eV.

When the activation energy of a given process is positive, it indicates that the process is benefited by increasing the temperature. On the other hand, when the activation energy is negative, the process is diminished by increasing the temperature. According to the analysis proposed by Revell and Williamson [31], the negative activation energy of the long disorder process involves a complex process that takes place in two stages, the first reversible and the second irreversible. Considering that the algorithm implemented in the simulations implies jumps that are not allowed, it is possible that these are directly related to the slow disorder process. It should be mentioned that when experiments are carried out to determine the degree of ordering of Ni_3_Al, both time constants have a positive value [27].

### 3.2. SRO Parameter Evolution

**Figure 7:**
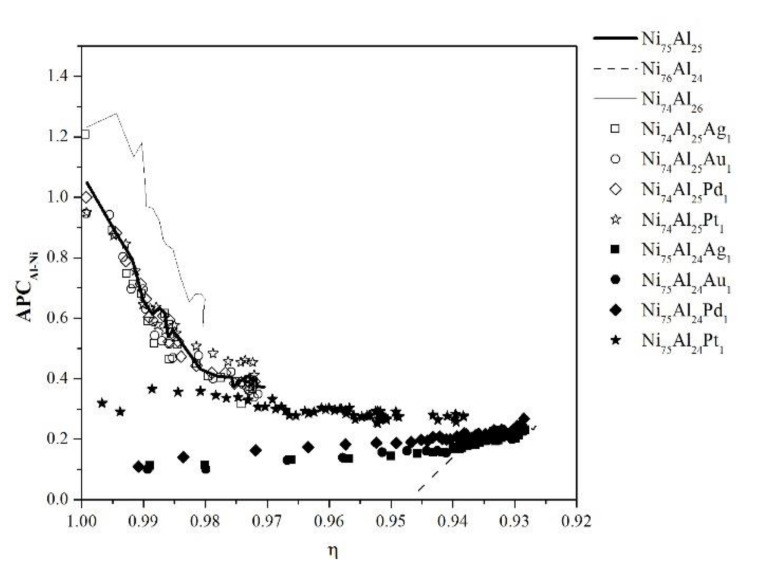
Antisite pair correlation parameter, *APC*_Al-Ni_.

Figure 7 shows the evolution of the *APC_Al-Ni_* parameter as a function of the ordering level (η) for all compositions at 1200 K. It is observed that in the samples with stoichiometric compositions Ni_75_Al_25_ and Ni_74_Al_25_X_1_, at the beginning of the process, the Ni antisites are surrounded by an Al antisite, and it decreases until reaching a value of *APC*_Al-Ni_ = 0.4; that is, there are 4 Al antisites for every 10 Ni antisites. The Ni_76_Al_24_ sample begins with a value of *APC*_Al-Ni_ = 0, and an ordering level of η = 0.947, which can be attributed to the level of Ni antisites with which it starts according to its stoichiometry. On the other hand, the samples with the Ni_75_Al_24_X_1_ formula show a value of *APC*_Al-Ni_ throughout the disordering process, which varies from 0.1 to 0.25, with the exception of the Ni_75_Al_24_Pt_1_ sample where the variation is from 0.32 to 0.28. The *APC*_Al-Ni_ evolution in the Ni_74_Al_26_ sample is similar to that of Ni_75_Al_25_, differing only by the magnitude and the equilibrium ordering level (η).

**Figure 8:**
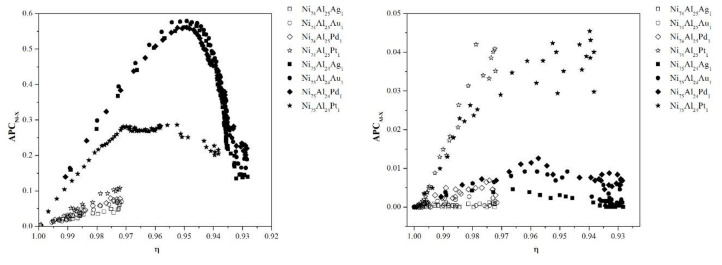

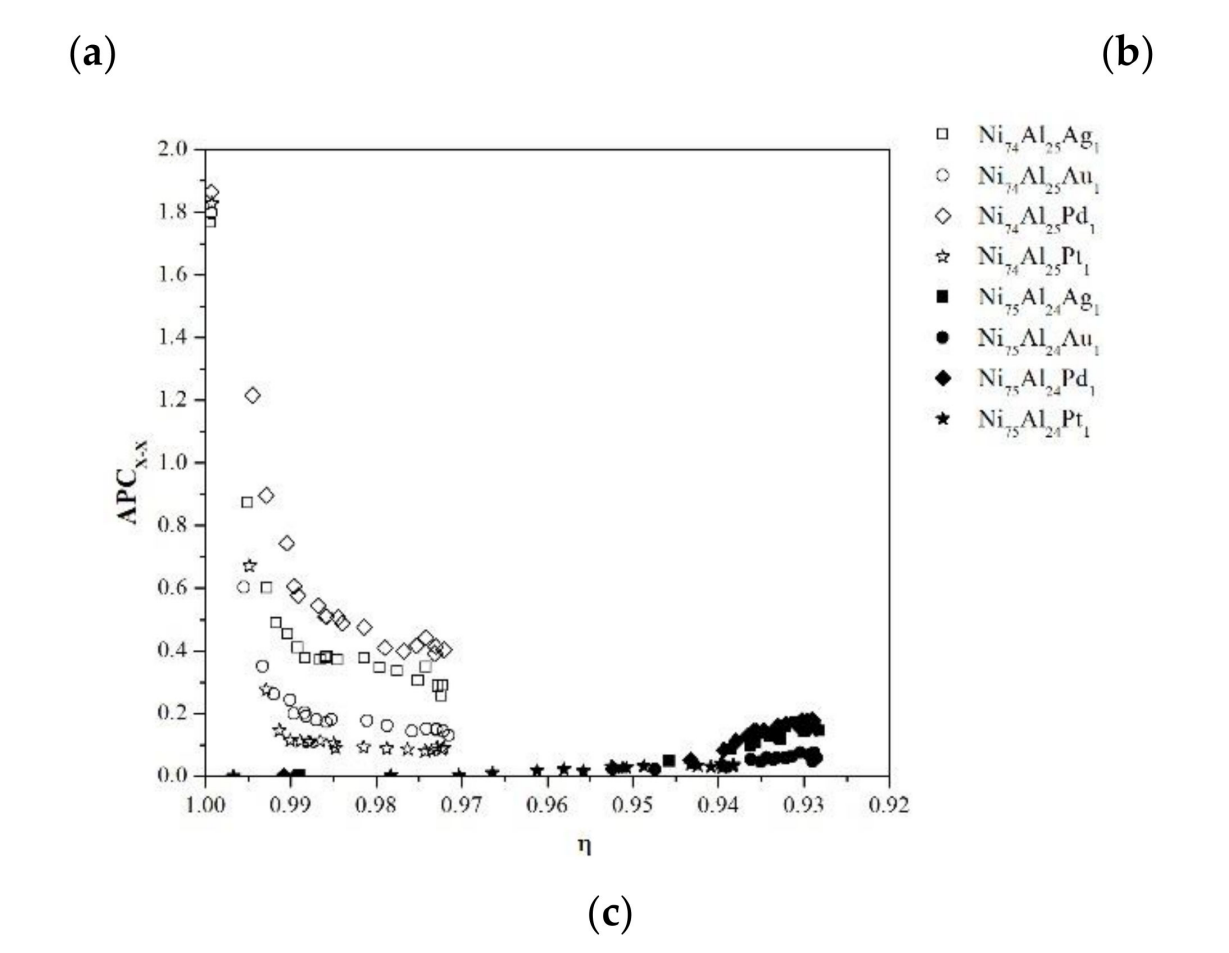
Correlation parameter of pairs of antisites involving noble metals: (**a**) *APC*_Ni-X_, (**b**) *APC*_Al-Ni_, and (**c**) *APC*_X-X_.

Figure 8 shows the evolution of the *APC* parameters (described in Equations (8) to (10)) as a function of the ordering level (η) for all compositions at 1200 K. Figure 8a represents the affinity of Ni antisites toward noble metals, starting in all cases from a value of *APC*_Ni-X_ = 0. In the samples with the Ni_74_Al_25_X_1_ formula, the increase in the *APC*_Ni-X_ parameter is linear and inversely proportional to the ordering level (η) during the whole disordering process, reaching equilibrium values of 0.05, 0.075, 0.076, and 0.1 for X = Ag, Au, Pd, and Pt respectively. On the other hand, in the samples with the Ni_75_Al_24_X_1_ formula doped with Ag, Au, and Pd, the affinity of Ni antisites toward noble metals has a parabolic behavior, reaching a maximum value of *APC*_Ni-X_ = 0.58 at an equilibrium level of η = 0.95, and subsequently decreases to a value of *APC*_Ni-X_ = 0.15. The Ni_75_Al_24_X_1_ sample shows a linear increase until it reaches a value of *APC*_Ni-X_ = 0.27 and an equilibrium level of η = 0.97, and from this point on, it remains constant and decreases when the equilibrium level is η = 0.965 to *APC*_Ni-X_ = 0.2.

Figure 8b shows the affinity of Al antisites toward noble metals located at Al sites. In general, it is observed that the affinity is minimal compared to that of Ni antisites. It is observed that the maximum affinity value corresponds to the samples doped with Pt (*APC*_Al-X_ = 0.04), while the other samples show a maximum value of *APC*_Al-X_ = 0.01.

Figure 8c shows the affinity of noble metal atoms for each other. It is observed that the samples with the Ni_74_Al_25_X_1_ composition have greater interaction with respect to those with the Ni_75_Al_24_X_1_ formulation. The interaction of dopants can be ordered as follows: Pd > Ag > Au > Pt.

### 3.3. Jump Profiles

**Figure 9:**
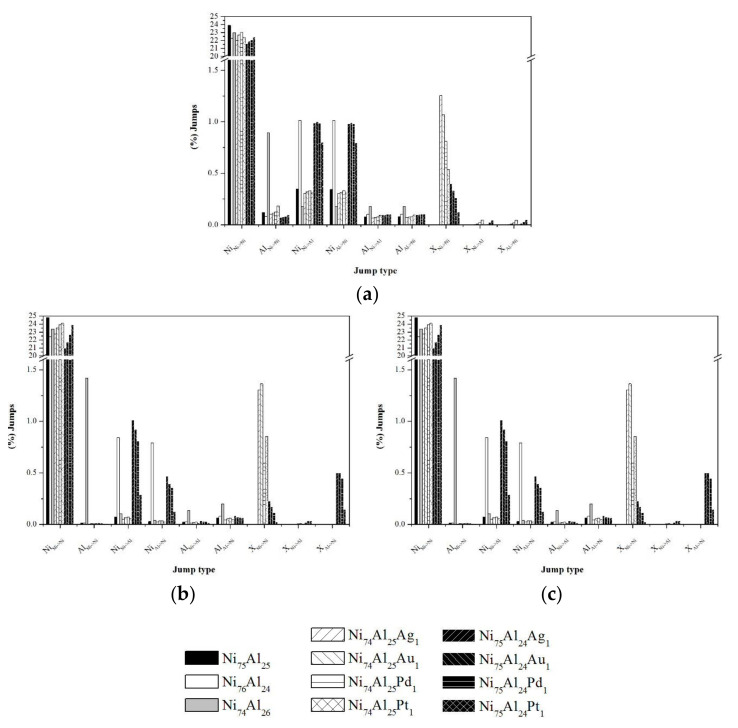
Jump statistics at 1200 K based on the atom and the direction it jumps: (**a**) at 50 × 10^6^ MCTime, (**b**) in the first 100,000 MCTime, and (**c**) in the last 100,000 MCTime.

A statistic of the percentages of jumps that promote the mobility of the vacancy was carried out considering the type of atom that jumps (Ni, Al, or noble metal “X”) and the direction in which it jumps. Figure 9a shows the results of all time steps of the simulations at 1200 K. It is observed that in all the samples, most of the jumps (22–24%) correspond to Ni jumps in their own sub-lattice (Ni_Ni__→Ni_). The other types of jump do not exceed 1.5%. There is a slight difference in the percentage of atomic jumps of Ni and Al with direction Ni-Al and Al-Ni (0.0040% and 0.0024%, respectively), which indicates an equilibrium condition; however, this can be attributed to the fact that the ordering level remained in equilibrium during a long simulation period. All the simulations presented in this work were performed at 50 × 10^6^ MCTime. This was sufficient in order to visualize the fall of the ordering level until reaching equilibrium. For example, in Figure 3, during the first 25 × 10^6^ MCTime it is observed that the ordering level reaches equilibrium and remains constant from that point until the 50 × 10^6^ MCTime is reached. In order to define the processes that exist during the start of the simulation, the jump statistics corresponding to the first 100,000 MCTime were obtained (Figure 9b). The results obtained indicate that there is a difference in the percentage of atomic jumps in the Ni-Al and Al-Ni direction, which is related to the rapid drop in the ordering level during the long process. The low percentage of Al jumps indicates that the limiting stage of the long disorder process is the Al_Al-Ni_ jump. Figure 9c presents the statistics for the last 100,000 MCTime of the simulation. It is observed that there is practically no difference between the atomic jumps in the Ni-Al and Al-Ni directions, indicating a state of equilibrium.

In the equilibrium of the Ni_74_Al_26_ sample, the mobility of Ni decreased and that of Al increased in both sub-lattices. This is related to the higher level of ordering that this sample has with respect to the stoichiometric sample. The Al mobility in the Ni sub-lattice is remarkable, being up to 8 times greater than those of the other samples. However, it is also important to note that in this sample, the mobility of Al and Ni in the Al sub-lattice is very similar. The increase in the Al jump statistic is due to the increase in the concentration of Al atoms in the Ni sub-lattice derived from the composition of the sample. Samples Ni_76_Al_24_ and Ni_74_Al_25_X_1_ show a remarkable mobility of Ni atoms through the Al sub-lattice; however, it is observed that the Pt-doped sample shows a lower frequency of mobility by 0.2%. In Ni_75_Al_24_X_1_ samples, the mobility of the noble metal atoms through the Ni sublattice stands out, maintaining the mobility of Ni and Al similar to that of the stoichiometric sample, Ni_75_Al_25_. In all doped samples, the noble metal mobility through the Ni sublattice is ordered as follows: Ag > Au > Pd > Pt.

**Figure 10:**
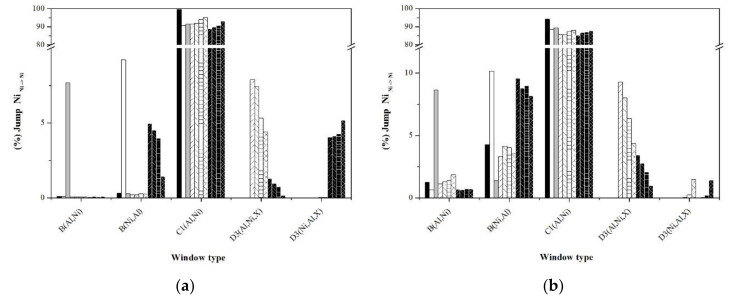
Statistics of Ni_Ni-Ni_ jumps typifying the windows: (**a**) First 100,000 Monte Carlo (MC)time of simulation, (**b**) last 100,000 MCTime of simulation.

**Figure 11:**
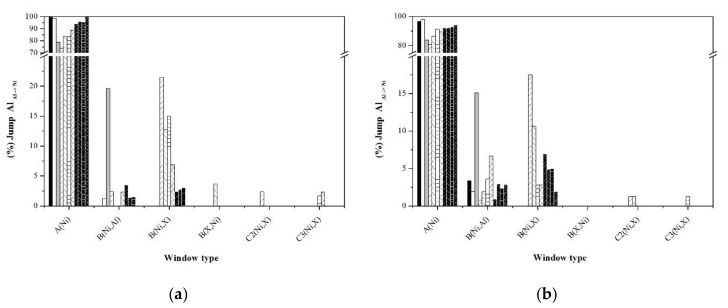
Statistics of Al_Al-Ni_ jumps typifying the windows: (**a**) First 100,000 MCTime of simulation, (**b**) last 100,000 MCTime of simulation.

**Figure 12:**
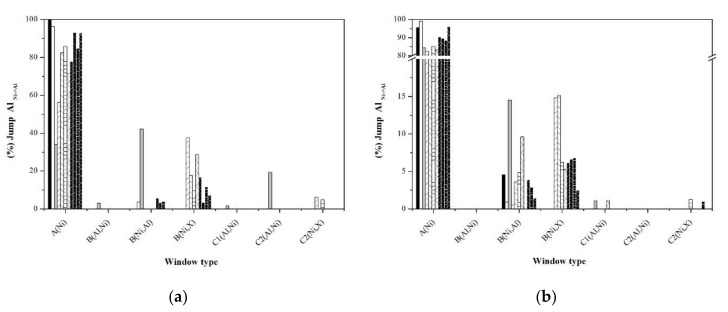
Statistics of Al_Ni-Al_ jumps typifying the windows: (**a**) First 100,000 MCTime of simulation, (**b**) last 100,000 MCTime of simulation.

**Figure 13:**
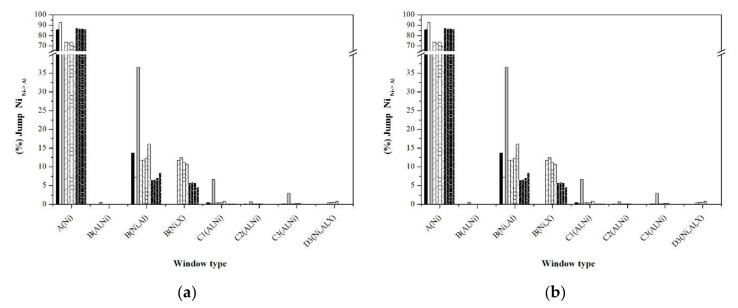
Statistics of Ni_Ni-Al_ jumps typifying the windows: (**a**) First 100,000 MCTime of simulation, (**b**) last 100,000 MCTime of simulation.

**Figure 14:**
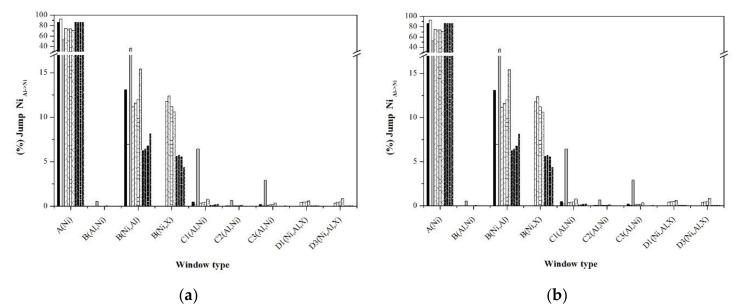
Statistics of Ni_Al-Ni_ jumps typifying the windows: (**a**) First 100,000 MCTime of simulation, (**b**) last 100,000 MCTime of simulation.

An alternative to analyze the short and long disorder processes separately is through the jump statistics, typifying them through the jump windows. Figure 10, Figure 11, Figure 12, Figure 13 and Figure 14 show the graphs that indicate the percentage of jumps based on the type of window they pass through. It should be mentioned that only the windows that showed a significant percentage of jumps are presented, which indicates their relevance in the disorder mechanism. Each figure shows the statistics of the jumps as a function of the windows through which they pass at the beginning and at equilibrium (the first and last 100,000 MCTime of the simulation). From the figures, it can be seen that in the Ni_Ni-Ni_, Al_Al-Ni_, and Al_Ni-Al_ jumps, there is a significant variation in the percentage of jumps between the initial stage of the simulation and that observed in equilibrium. In the initial stage, jumps in “natural” windows prevail, while in equilibrium, there is an increase in the percentage of jumps through “unnatural” windows.

The frequency of Ni_Ni-Ni_ jumps in the stoichiometric sample takes place mainly in the natural window C1(Al,Ni), followed to a lesser extent by windows B(Ni,Al) and B(Al,Ni), whose configuration obeys the state of localized disorder. The nonstoichiometric formulations promote the formation of type B windows and the type Ni_Ni-Ni_ jumps through them. As a consequence, this causes the decrease in the percentage of this type of jump in the “natural window” C1(Al, Ni). On the other hand, noble metals promote the Ni_Ni-Ni_ jumps mainly through type D3(Al,Ni,X) windows, and the influence on the percentage of jumps according to the dopant noble metal is Ag > Au > Pd > Pt. In particular, in the Pt-doped samples, Ni_Ni-Ni_ jumps are carried out through the D3 window (Ni,Al,X).

According to Figure 11b, in equilibrium, the Al_Al-Ni_ jumps are carried “natural window” A(Ni), followed by the window B(Ni,X) (influenced by the type of noble metal dopant; Ag > Au > Pd > Pt), and finally from window B(Ni,Al). On the other hand, the Ni_Ni-Al_ and Ni_Al-Ni_ jumps have practically the same percentage both at the beginning of the simulations and at equilibrium, which indicates the independence of this type of jump throughout the disordering process. The above suggests that the jumps that govern the disordering process are the Ni_Ni-Ni_ jumps through which the Ni vacancy transits in the entire arrangement of atoms, and once it is in the vicinity of an Al atom, the jump Al_Al-Ni_ originates an Al vacancy, which is immediately occupied by an Ni atom through a jump Ni_Ni-Al_, whose percentage of jumps is constantly higher.

**Figure 15:**
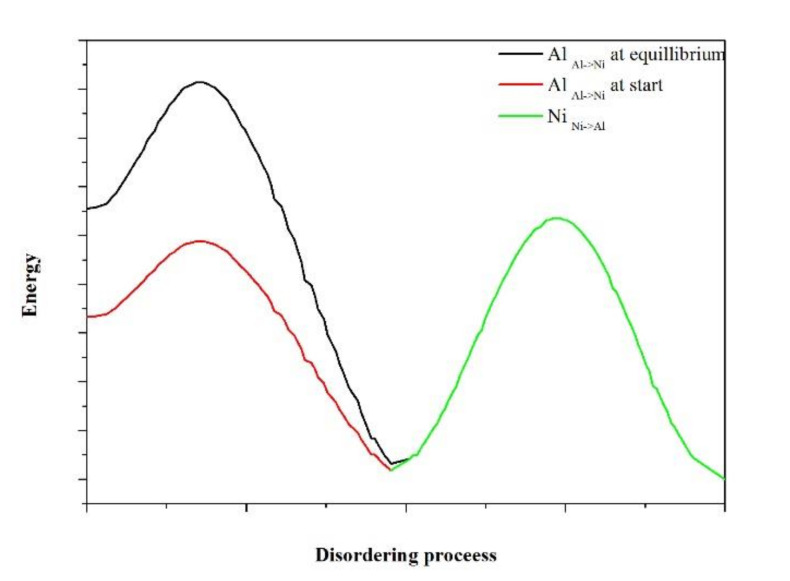
Interpretation of the origin of the time constants of the short and long ordering processes from the point of view of the energy of the Al_Ni-Al_ and Ni_Al-Ni_ jumps.

Revell and Williamson [31] carried out a rigorous analysis of the enthalpy and entropy aspects that characterize the sub-processes that are part of a general process with a negative activation energy, such as the activation energy obtained considering the time constant of the slow ordering process which can be interpreted from the point of view of the energy of the processes as shown in the diagram in Figure 15. The short clutter process consists of Al_Al-Ni_ jumps through natural windows whose energy requirement is low [20]. On the other hand, the long clutter process consists of Al_Al-Ni_ jumps through unnatural windows that have a higher energy cost, while in both cases, the energy level necessary for the Ni_Ni-Al_ jumps is constant during both processes of disorder. The change in the activation energies in the samples doped with noble metals is related to a change in the energy of the Al_Al-Ni_ jumps and, according to the statistics presented (Figure 11 and Figure 12), indicate a very important role of the window B(Ni,X).

## 4. Conclusions

Based on the Monte Carlo simulations of the Ni_3_Al intermetallic disorder process, in its stoichiometric and nonstoichiometric state and also doped with noble metals (Ag, Au, Pd, and Pt) at temperatures below the disorder temperature, the following conclusions were obtained:

The simulation results made it possible to define the activation energies of the short and long disorder processes. In addition, jump statistics were generated, typifying them according to the type of atom, direction, and window configuration in which the jump was made.

Nonstoichiometric and noble metal-doped formulations show a change in activation energy in the short and long disorder processes, which implies a variation in the energy of the jumps that generate disorder. According to the statistics, it was determined that the percentage of Ni_Ni-Ni_, Al_Ni-Al_, and Al_Al-Ni_ jumps shows a difference between the initial stage of the simulation and that of equilibrium. 

The analysis of the energy of the jumps allowed the definition of the origin of the negative activation energy obtained from the long disordered time constants, which is due to an increase in the energy of the Al_Al-Ni_ jumps due to the unnatural windows generated during the disordering process. Furthermore, the energy of the Ni_Ni-Al_ jumps can be considered constant as the statistics of this type of jump do not change between the initial stage of the simulation and that of equilibrium.

The deviations in the ordering level during the simulation of samples doped with noble metals are related to the stability that each noble metal atom has when it is substituted in an Ni or Al site. Such is the case of the Pt-doped samples, whose atom is stabilized at both the Ni and Al sites, which results in a high ranking level with respect to the other noble metals, which tend to preferentially stabilize at the Ni sites.
